# Antimicrobial Resistance in Class 1 Integron-Positive Shiga Toxin-Producing *Escherichia coli* Isolated from Cattle, Pigs, Food and Farm Environment

**DOI:** 10.3390/microorganisms6040099

**Published:** 2018-09-28

**Authors:** Rocío Colello, Alejandra Krüger, José Di Conza, John W. A. Rossen, Alexander W. Friedrich, Gabriel Gutkind, Analía I. Etcheverría, Nora L. Padola

**Affiliations:** 1 Laboratorio de Inmunoquímica y Biotecnología, Facultad de Ciencias Veterinarias, UNCPBA 7000, Argentina; akruger@vet.unicen.edu.ar (A.K.); analiain@vet.unicen.edu.ar (A.I.E.); nlpadola@vet.unicen.edu.ar (N.L.P.); 2 Centro de Investigación Veterinaria de Tandil (CIVETAN), CONICET, CICPBA 7000, Argentina; 3 Laboratorio de Resistencia Microbiana, Cátedra de Microbiología, Facultad de Farmacia y Bioquímica, UBA, Buenos Aires 1426, Argentina; jdiconza@gmail.com (J.D.C.); ggutkind@ffyb.uba.ar (G.G.); 4 Department of Medical Microbiology, University Medical Center Groningen, University of Groningen, 9713 GZ Groningen, The Netherlands. j.w.a.rossen@rug.nl (J.W.A.R.); alex.friedrich@umcg.nl (A.W.F.)

**Keywords:** antimicrobial resistance, class 1 integron, STEC, reservoirs

## Abstract

The aim of this study was to investigate the presence of class 1 integrons in a collection of Shiga toxin-producing *Escherichia coli* (STEC) from different origins and to characterize pheno- and genotypically the antimicrobial resistance associated to them. A collection of 649 isolates were screened for the class 1 integrase gene (*intI1*) by Polymerase chain reaction The variable region of class 1 integrons was amplified and sequenced. Positive strains were evaluated for the presence of antimicrobial resistance genes with microarray and for antimicrobial susceptibility by the disk diffusion method. Seven out of 649 STEC strains some to serogroups, O26, O103 and O130 isolated from cattle, chicken burger, farm environment and pigs were identified as positive for *intl1*. Different arrangements of gene cassettes were detected in the variable region of class 1 integron: *dfrA16*, *aadA23* and *dfrA1-aadA1*. In almost all strains, phenotypic resistance to streptomycin, tetracycline, trimethoprim/sulfamethoxazole, and sulfisoxazole was observed. Microarray analyses showed that most of the isolates carried four or more antimicrobial resistance markers and STEC strains were categorized as Multridrug-resistant. Although antimicrobials are not usually used in the treatment of STEC infections, the presence of Multridrug-resistant in isolates collected from farm and food represents a risk for animal and human health.

## 1. Introduction

Shiga toxin-producing *Escherichia coli* (STEC) constitute a significant group of emerging enteric pathogens. STEC cause foodborne illnesses worldwide that result in life-threatening complications such as hemolytic-uremic syndrome (HUS) and hemorrhagic colitis (HC) [1]. The most important virulence factors associated with STEC infection are the potent cytotoxins: Stx1 and Stx2, encoded by *stx1* and *stx2* genes, respectively [2]. The first step in STEC pathogenesis is the capacity to compete with commensal microbiota and colonize the human intestine [3]. The ability to adhere to epithelial cells is an important virulence trait, since it presumably allows toxins to be efficiently delivered to the host organs [4]. STEC can produce intimin (an adherence membrane protein which is required for the attachment to the host intestinal mucosa), an autoagglutinating adhesion protein (Saa), a plasmid-encoded enterohemolysin (EhxA), a catalase-peroxidase (KatP), a subtilase cytotoxin (SubAB), a virulence factor that contributes in hemagglutination, adhesion and autoaggregation (Hes), among others [5,6,7,8,9].

Cattle, the main reservoir of STEC, contaminated food and farm environment contribute to the transmission to humans. STEC strains isolated from different sources possess a great variety of virulence encoding genes. In the last few years, antimicrobial resistance has been described in STEC strains [10,11,12,13]. Although antibiotic treatment is not recommended in STEC infection, these strains can transfer the resistant genes to other bacteria. The emergence and dissemination of resistant bacteria have been related to overuse and misuse of antimicrobials. The antimicrobials resistance can occur spontaneously by mutation or by horizontal gene transfer (HGT) from relatives or from nonrelatives bacteria by mobile genetic elements such as plasmids, transposons and integrons [14]. 

Integrons are genetic elements that can acquire exogenous gene cassettes (usually antimicrobial resistance genes). Thus, many resistance cassettes can be recruited by integrons and thereby enhance the bacterium antimicrobial resistance [15]. Class 1 integrons represent the most common structure among the integrons, and they are characterized by the presence of two conserved segments. The 5’ conserved segment encodes an integrase (*intl1*) and contains a strong promoter that enables expression of the integrated cassettes. Integrase gene is potentially indicative of the ability to recruit antimicrobial resistance genes [16,17]. The commonest 3’ conserved segment (3’CS) carries *qac*EΔ1 that encodes for resistance to antiseptics and disinfectant, the *sul1* gene (confers sulfonamide resistance), and an open reading frame—orf5 of unknown function [18,19].

The misuse of antimicrobial agents in agriculture or veterinary could contribute to the dissemination of resistance genes among bacteria. Therefore, taking into account the hazard of antibiotic-resistance genes in STEC and the possibility to be transferred to other bacteria, the aim of this study was to investigate the presence of class 1 integrons in a large collection of STEC isolates from different origins and to characterize pheno- and genotypically the antimicrobial resistance associated to class 1 integron-positive isolates. 

## 2. Materials and Methods

### 2.1. Bacterial Strains

A collection of 649 STEC (from cattle (*n* = 456), pigs (*n* = 50), food (*n* = 85), farm environment (*n* = 34) and chicken burger (*n* = 24)) was investigated. The isolates were obtained between 2009 and 2015 from Argentina, were analyzed for the presence of *stx1*, *stx2*, *eae*, *ehxA* and *saa* genes, and were serotyped [11,20,21,22,23].

### 2.2. PCR Detection of intl1 Gene

To identify isolates carrying the class 1 integron, the presence of *intI1* was evaluated by PCR [24]. DNA was obtained by boiling bacteria suspended in sterile water for 10 min as described previously by Parma, et al. [25]. The PCR reaction was performed in a total volume of 50 µL using a T-17 thermal cycler (Ivema, Argentina). Initial denaturation at 94 °C for 10 min was followed by 30 cycles of denaturation at 94 °C for 1 min, primer annealing at 57 °C for 1 min, extension at 72 °C for 2 min; with a final extension at 72 °C for 10 min. Amplification products were separated by electrophoresis on 2% agarose gels containing 0.8 µg/mL of ethidium bromide (Inbio Highway, Tandil, Argentina) in running buffer and were visualized by a UV transilluminator (Vilber Lourmat, Marne La Velle, France).

### 2.3. Arrangement of Resistance Gene Cassettes in Class 1 Integrons 

PCR for variable regions was carried out using the following primers: 5′CS: 5′-GGCATCCAAGCAGCAAG and 3′CS: 5′-AAGCAGACTTGACCTGA (Di Conza et al., 2002). PCR products were analyzed on 1% agarose gel. Amplification products were obtained from the gels with the DNA PuriPrep-GP kit (INBIO HIGHWAY, Argentina). The amplified products were sequenced at Macrogen with the class 1 variable region primers (5′CS and 3′CS). Sequences were compared with the GenBank database using the BLASTN or BLASTX program available at the National Center for Biotechnology Information website (www.ncbi.nih.gov) and the INTEGRALL database (http://integrall.bio.ua.pt/).

### 2.4. Antimicrobial Susceptibility Testing

Antimicrobial susceptibility profiles in class 1 integron-positive STEC strains were determined by the disk diffusion method according to the Clinical and Laboratory Standards Institute guidelines [26]. The following antimicrobials were assayed: ampicillin (AMP 10 μg), gentamicin (GEN 10 μg), amikacin (AKN 30 μg), kanamycin (KAN 30 μg), tetracycline (TET 30 μg), nalidixic acid (NAL 30 μg), ciprofloxacin (CIP 5 μg), chloramphenicol (CMP 30 μg), trimethoprim/sulfamethoxazole (TMS 1.25/23.75 μg), sulfisoxazole (SOX 300 μg) and streptomycin (S 10 μg) [27]. Multidrug-resistant bacteria (MDR) were defined as strains resistant to three or more antimicrobial families [28].

### 2.5. Antimicrobial Resistance Genes

Genomic DNA was obtained using the UltraClean Microbial DNA Isolation Kit (Mo Bio, Carlsbad, CA, USA) or Wizard Genomic DNA Purification Kit (Promega, Madison, WI, USA) according to the manufacturer’s instructions. The presence of antimicrobial resistance genes was evaluated with a commercial oligonucleotide microarray for *E. coli* according to the manufacturer’s protocol (CLONDIAG *Escherichia coli* combined Assay, Alere Technologies GmbH, Jena, Germany) [29]. Arrays contained 102 cDNA probes targeting antimicrobial resistance genes. Visualization of hybridization was achieved using the ArrayMate instrument and signals were analyzed automatically. 

## 3. Results 

Seven out of 649 STEC strains (1.07%), some to serogroups O26, O103 and O130 isolated from cattle, chicken burger, farm environment and pigs were identified as positive for *intl1* (Table 1). PCR amplification and DNA sequencing of the variable region of class 1 integrons showed amplicons of different sizes. STEC O130:H21 carried a 0.75 kb amplicon containing a *dfrA16* cassette encoding resistance to trimethoprim. A 1.0 kb amplicon containing an *aadA* cassette, which encodes resistance to streptomycin-spectinomycin, was detected in two strains isolated from pigs, while a 1.5 kb amplicon containing *aadA* and *dfrA1* genes was obtained from four STEC isolated from bovine and farm environment. The resistance patterns of STEC isolates are shown in Table 1. In most of the 7 isolates evaluated by DNA microarray carried four or more antimicrobial resistance markers (Table 1). All isolates carried one or more sulfonamide resistance gene(s) (*sul1* and/or *sul2*), while 5/7 (71%) of STEC strains were positive for some of the tetracycline resistance genes (*tetA* or *tetB*) and 5/7 for *bla*_TEM_ genes. Aminoglycoside resistance (*aadA* alone or in combination with *strA*-*strB* and *aphA*), trimethoprim resistance (*drfA1*) and chloramphenicol resistance (*catA1* or *floR*) were also detected. The phenotypic resistance profiles of two strains could not be fully explained by the microarray results. The first one was the resistance to tetracycline observed in STEC O103:H2, and the other one was the resistance to trimethoprim in STEC O130:H11. The latter could be explained by integron sequencing that revealed the presence of *drfA1*6, not identified with the probes included in the array. Moreover, most genetic resistant profiles were phenotypically confirmed. Six (86%) isolates showed resistance to tetracycline, streptomycin, and chloramphenicol, 5 (71%) to trimethoprim/sulfamethoxazole, 5 (71%) to sulfisoxazole (and the remaining 2 showed intermediate susceptibility), 5 (71%) to ampicillin (and 1 showed intermediate susceptibility), and 4 (57%) to nalidixic acid. One isolated showed intermediate susceptibility to kanamycin.

## 4. Discussion

The present study evaluated the antimicrobial resistance and the molecular profile in class 1 integron-positive STEC belonging to serogroups associated with cases of diarrhea and HUS in the world [30]. It is noteworthy that from a large collection of STEC strains isolated from animals and food, class 1 integrons were detected at a low rate. Other studies also reported strains from different sources that have acquired integrons and antimicrobial resistance genes, including STEC O26, O103, O111 [13,19,31,32,33]. 

Three different arrangements of gene cassettes were detected in the variable region of class 1 integron: *dfrA16*, *aadA23*, and *dfrA1*-*aadA1*. The latter combination, encoding resistance to trimethoprim and streptomycin respectively, has been reported previously in non-O157 STEC isolates [31,34]. Contrary to our results, Chen et al. [33] found *aadA1*, *dfrA1* and *arr*-3 arrangement gene cassette and Maynard, et al. [35] found *dfrA1*, *dfrA5* and *dfrA1*. Some studies showed isolates carrying integrons and resistance to two or more classes of antimicrobials supporting the hypothesis of an association between the presence of class 1 integrons and emerging MDR [36] in agreement with this work in which all strains carrying integrons were classified as MDR. However, integrons and their gene cassettes did not always account for the total resistance exhibited by the isolates [19]. 

STEC constitute one of the most important causes of foodborne diseases. Currently, the best management of patients with STEC infections includes close monitoring to avoid the development of complications in combination with supportive therapy [37]. Antimicrobial treatment for STEC infections is controversial [38] and it is generally not recommended, but the indirect selection for multiresistant strains could occur due to the antibiotic induced selection pressure on other diseases causing bacteria. In addition, antimicrobials are widely used prophylactically, metaphylactically and as growth promoters in animal husbandry [39]. Growth promoters belonging to different groups of antimicrobials structurally unrelated exert their antibacterial activity by several mechanisms. 

In our study, resistance to β-lactams, tetracycline and trimethoprim/sulfamethoxazole was observed in most of the isolates. The β-lactamase gene TEM-1 (*bla*_TEM-1_) is the most prevalent β-lactamase in gram-negative bacteria which is usually located on conjugative plasmids facilitating its spread among different species [40]. Some authors reported *bla*_TEM-1_ in STEC, not only isolated from food or animals but also from humans [13,40]. We observed high rates of resistance to tetracycline and trimethoprim-sulfamethoxazole that is in agreement with others studies conducted in STEC [19,41]. All tetracycline resistant isolates harbored *tetA*, and/or *tetB*. Tetracycline has been used worldwide in both human and veterinary medicine due to being able to select resistance strains [35]. All isolates were positive for one or more sulfonamide resistance genes *sul1* and/or *sul2*. This result agrees with the fact that the *sul1* gene is part of the 3′CS in class 1 integron. The sulphonamide resistance genes may be present in diverse mobile genetic elements (such as integrons) that can be easily disseminated to other bacteria. The use of this antimicrobial in veterinary medicine or food animals may contribute to their maintenance of *Escherichia coli* strains [42].

## 5. Conclusions

In summary, class 1 integrons were detected in a low proportion in STEC strains isolated from food, animals and farm environment of our region. Integrons and their associated gene cassettes did not always account for the whole phenotypic resistance profile exhibited in these isolates. Other genetic platforms than class 1 integrons and their associated gene cassettes contribute to the antimicrobial resistance phenotype. Although antimicrobials are not usually used in the treatment of STEC infections, the presence of MDR in isolates collected from farm and food represents a risk for animal and human health as they can spread their resistance genes to other bacteria. We conclude that class 1 integrons may contribute to the emergence and dissemination of antimicrobial resistance among STEC in humans, food, and animals, therefore, some measures must be taken to ensure a reasonable use of antimicrobials in the animal husbandry.

## Figures and Tables

**Table 1:** Antibiotic resistance phenotype and genotype in class 1 integron-positive isolates.

Pathotype	Year of Isolation	Origin	Serogroup/Serotype	Virulence Marker	Resistance Phenotype	Antibiotic Resistance Genes by Microarray	Class 1 Integron	
							Amplicon size	Antibiotic Resistance Gene Cassettes
STEC	2012	chicken burger	O130:H11	*stx1, stx2, ehxA, saa*	TMS-NAL-TET-SOX-amp-cip-gen *	*sul1, tetA*	0.75 kb	*dfrA16*
STEC	2009	farm environment	ONT:H18	*stx1, ehxA, eae*	AMP-CMP-TMS-TET-NAL-S-sox *	*sul2, tetB, strA/B, aadA1, blaTEM, floR, dfrA1*	1.5 kb	*dfrA1-aadA1*
STEC	2009	Bovine	O103:H18	*stx1, eae, ehxA*	AMP-CMP-TMS-S-sox *	*sul1/2, strA/B, aadA1, blaTEM, floR, dfrA1*	1.5 kb	*dfrA1-aadA1*
STEC	2009	Bovine	O103:H2	*stx1, eae, ehxA*	AMP-CMP-TMS-TET-SOX-S	*sul1/2, strA/B, aadA1, blaTEM, floR, dfrA1, ‡*	1.5 kb	*dfrA1-aadA1*
STEC	2009	Bovine	O26:H11	*stx2, eae, ehxA*	AMP-CMP-TMS-TET-SOX-S	*sul1/2, tetA, strA/B, aadA1, blaTEM, floR, dfrA1*	1.5 kb	*dfrA1-aadA1*
STEC	2016	Pig	O2:H32	*stx2*	CMP-NAL-TET-SOX-S	*sul1, tetB, aadA1, catA1*	1 kb	*aadA23*
STEC	2016	Pig	ONT:H32	*stx2*	AMP-CMP-NAL-TET-SOX-S-kan *	*sul1, tetB, aadA1, blaTEM, catA1*	1 kb	*aadA23*

* phenotypes written in lowercase indicate intermediate susceptibility; ‡ ambiguous result for tetA/B/C; AMP (Ampicillin), CIP (ciprofloxacin), CMP (Chloramphenicol), GEN (gentamicin), KAN (kanamycin), NAL (Nalidixic acid), S (streptomycin), SOX (sulfisoxazole), TET (Tetracycline), TMS (Trimethoprim/Sulfamethaxazole).
